# COVID-19 in Princess Marina Hospital, Botswana: An Outbreak Investigation

**DOI:** 10.1155/2022/2663174

**Published:** 2022-04-12

**Authors:** Keatlaretse Siamisang, Dineo Kebadiretse, Pamela Smith-Lawrence

**Affiliations:** ^1^Department of Family Medicine and Public Health, University of Botswana, Gaborone, Botswana; ^2^Department of Health Services Management, Botswana Ministry of Health and Wellness, Gaborone, Botswana

## Abstract

The Princess Marina Hospital in Gaborone, Botswana, had an outbreak of COVID-19 from early August 2020. The aim of this paper was to describe the outbreak investigation. The investigation's specific objectives were to describe the COVID-19 cases in terms of person, place, and time (PPT) and to determine measures to prevent further transmission of the infection. The data reported herein were collected over a 3-month period from beginning of August to end of October 2020. The investigation included all COVID-19 cases i.e. both patients and healthcare workers. It followed the steps of an outbreak investigation. These included assembling an investigation team comprising both the hospital and DHMT staff. All the wards reported their confirmed cases to the infection control team who in turn prepared line lists and case reports. Epicurves were produced from date of positive result. A total of 193 cases were reported, of which 110 (57.0%) were patients and 83 (43.0%) were healthcare workers. The median age was 35 years. Females accounted for 154 (79.8%) participants. Most of the wards were affected. The wards with the highest numbers of cases were female medical ward (39), emergency department (24), gynecology ward (17), and pediatric medical ward (10). Control measures included restricting movement into the hospital as well as clinical screening at all entry points. Furthermore, all patients were tested before admission into the wards. Surveillance of COVID-19 cases was continued beyond the 3 months reported in this paper. COVID-19 can spread rapidly in hospital settings affecting both patients and healthcare workers. Outbreak investigations including describing cases in terms of person, place, and time are critical if the most effective and efficient control measures are to be implemented.

## 1. Introduction 

A cluster of patients presenting with pneumonia of unknown origin was reported in Wuhan, China, towards the end of 2019 [1]. This was later found to be caused by a double-stranded RNA, later named severe acute respiratory syndrome coronavirus-2 (SARS-CoV-2), and the disease was named COVID-19 [1]. The virulence of the respiratory virus made its transmission across the globe very rapid with the World Health Organization (WHO) consequently declaring it a Public Health Emergency of International Concern (PHEIC) in January 2020 [2]. Botswana declared its first confirmed case in March 2020, which was followed by a steady increase in imported cases throughout that year [3]. The government implemented several measures to manage the epidemic including setting up a national taskforce and restricting travel into and out of the country [4]. Princess Marina Hospital (PMH) had multiple and ongoing positive cases of COVID-19 affecting both patients and staff from early August 2020. The first documented confirmed case in this outbreak was diagnosed on 5^th^ August 2020. This was a resident foreign truck driver who works locally and was a contact of a positive case from a local school. Following this, a female patient in female medical ward who received dialysis care at a private renal care facility was diagnosed on 9^th^ August 2020. Contact tracing and testing of close contacts revealed multiple contacts. An investigation team from the Gaborone District Health Management Team (DHMT) worked with the hospital Rapid Response Team (RRT) to conduct an outbreak investigation. Active surveillance was initiated. Among other things, the hospital infection control team wrote case summaries of all the positive cases in the hospital. The case summaries were based on interviews with the affected persons. This information was shared with the hospital management and the DHMT. They also prepared line lists of all the COVID-19 infected persons.

There is currently limited information on investigations of outbreaks in different health facilities across Botswana despite the additional value, this epidemiological scrutiny may add to the current COVID-19 response. The growing concern is that with confirmed cases increasing within the community, the probability of outbreaks in hospitals is inevitable. It is therefore pertinent to continually assess outbreaks within health facilities to strengthen measures aimed at the protection of healthcare workers in the workplace as well as disrupting the transmission chain. It is therefore undeniable that this high-risk biological hazard needs to be prevented and controlled within hospital settings where the risk of transmission is higher than that in the community. This is due in part to exposure to aerosol-generating procedures [5].

An outbreak investigation was therefore done to evaluate COVID-19 cases in PMH in Gaborone and to determine effective prevention and control measures that are appropriate within the hospital setting. The aim of this paper was to describe the COVID-19 outbreak investigation in PMH.

## 2. Methods and Findings

### 2.1. Study Site

This investigation was conducted in PMH in Gaborone, the capital city of Botswana. PMH is one of the 3 referral hospitals in the country. It is the busiest and largest hospital in the country with a capacity of 567 beds and an average of 750 inpatients.

### 2.2. Study Population

The study population was all COVID-19 cases in PMH during the study period. This included all affected patients and healthcare workers.

### 2.3. Study Design

**Figure 1:**
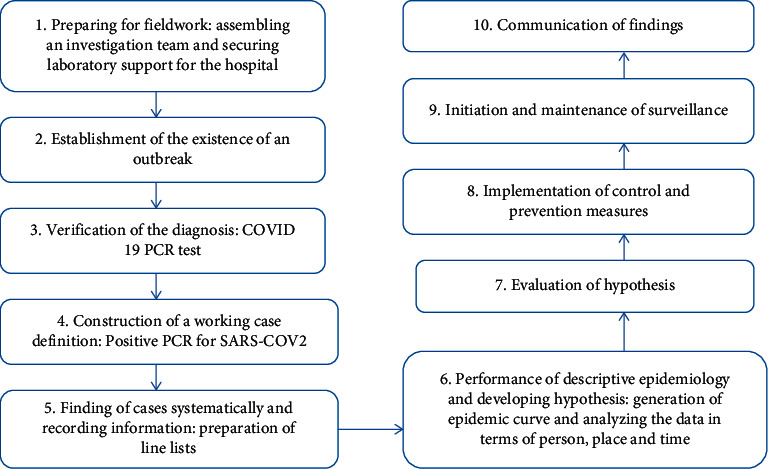
Steps of the outbreak investigation.

The investigation in PMH followed the 10 steps of an outbreak investigation. The methods and findings of this paper will follow these steps and not the convectional research format [6]. The 10 steps are shown in the flowchart (Figure 1).

#### 2.3.1. Prepare for Fieldwork

The preparation for fieldwork included assembling an investigation team and securing laboratory support for the hospital. The hospital RRT led the investigation. This comprises the hospital senior management and the infection control team. The infection control team is an integral part of the RRT in PMH. It is tasked with leading the hospital Infection Prevention and Control (IPC) initiatives including ensuring that hospital units are compliant with IPC protocols. This includes carrying out of standard type audits in the wards. The infection control team also worked with Gaborone DHMT to make an assessment of the hospital's readiness and compliance with COVID-19 protocols. A team from Gaborone DHMT was also deployed to support the investigation. This team comprised public health medicine residents and a community health nurse.

#### 2.3.2. Establish the Existence of an Outbreak and Verify the Diagnosis

At the time of the investigation, an outbreak of COVID-19 in the hospital was defined as diagnosis of COVID-19 in persons who would otherwise not be expected to have the infection. Therefore, after confirming the first few cases, an outbreak was promptly declared. The diagnosis was confirmed with a COVID-19 polymerase chain reaction (PCR) test.

#### 2.3.3. Construct a Working Case Definition

The case definition was according to the World Health Organization (WHO) definition of a confirmed case. In this definition, a confirmed case is a person with a positive Nucleic Acid Amplification Test (NAAT). All the cases during this investigation were confirmed with a COVID-19 PCR test.

#### 2.3.4. Find Cases Systematically and Record Information

All the wards reported their confirmed cases to the infection control team which was part of the RRT. The team then prepared line lists and case reports using the patient information. Variables captured included the demographics, date of testing, and date of confirmation. We report on data collected over a 3-month period from beginning of August 2020 to end of October 2020. At the time of the investigation, most of the confirmed cases were transferred to a designated COVID-19 hospital so the outcomes were not verified for most patients.

#### 2.3.5. Perform Descriptive Epidemiology and Develop Hypothesis

The data from the line lists and case reports were captured in an excel spreadsheet and were analyzed. IBM Statistical Package for the Social Sciences (SPSS version 24) and Epi info™ software version 7.1(2019) were used for data analysis. Categorical data were summarized with frequencies and percentages while numeric data were summarized with medians and interquartile ranges. Date of positive test was used to produce an epicurve of the outbreak.

Table 1 presents the characteristics of the participants. A total of 193 people at PMH were diagnosed with COVID-19 in the 3-month period. Of these, 110 (57.0%) were patients and 83(43.0%) were healthcare workers. Their age range was 0 to 86 years, and the median age was 35 years. Females accounted for 154 (79.8%) of all participants. Most of the hospital wards were affected. The wards with the highest numbers of confirmed cases were female medical ward (39), emergency department (24), gynecology ward (17), and pediatric medical ward (10).

The COVID-19 cases were stratified according to whether they are patients or healthcare workers in Table 2. The age range was 0 to 86 years for the patients and 19 to 63 years for the healthcare workers. Females accounted for 79.1% of all the patients and 80.7% of the healthcare workers. Female medical ward had the highest number of patient and healthcare worker cases.

**Figure 2:**
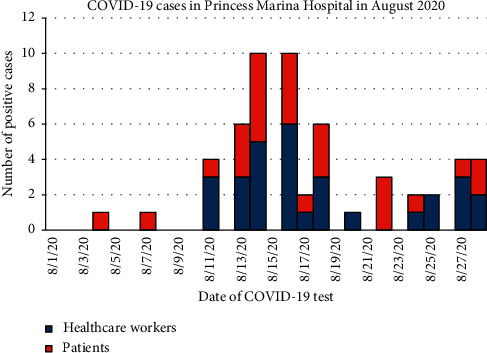
Epicurve for COVID-19 cases at Princess Marina Hospital in August 2020.

Figure 2 shows the epicurve for the COVID-19 cases stratified by whether they were patients or healthcare workers in the first month of the outbreak. The first case was a patient who was tested on the 4^th^ August 2020 followed by another patient on the 7^th^ August 2020. The first 3 healthcare workers were diagnosed on the 11^th^ August 2020 with a peak of 6 cases on the 16^th^ August 2020. The patient cases reached a peak of 5 on the 14^th^ August 2020.

**Figure 3:**
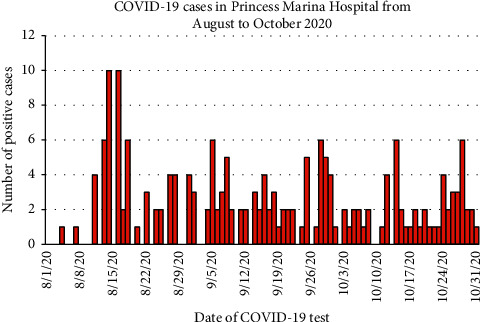
Epicurve for COVID-19 cases in Princess Marina Hospital from August to October 2020.

Figure 3 shows the epicurve for all COVID-19 cases in PMH from August 2020 to end of October 2020. After a peak of 10 on the 14^th^ and 16^th^ of August, the daily cases dropped to up to 6 cases per day for the 3-month period.

The outbreak affected patients and healthcare workers throughout the hospital. It was hypothesized that transmission of COVID-19 in the wards was a result of a breach of infection prevention and control protocols. It was also hypothesized that the infections were a result of community transmission of COVID-19.

#### 2.3.6. Evaluate the Hypothesis

Standard-type audits were conducted in various wards. These were designed mainly to assess the hospital COVID-19 structures and processes. These investigations will be discussed in detail in other publications.

#### 2.3.7. Implement Control and Prevention Measures

Control measures included restricting movement into the hospital. Entry to the hospital was limited to patients and staff. Screening including temperature checks was done at all hospital entry points, and all patients were tested before admission into the wards.

The following recommendations were made to limit further spread of COVID-19 in the hospital wards:

1. The hospital should have an isolation ward with dedicated staff. There should be designated donning and doffing areas and well-defined patient and staff flow in the isolation wards.
2. The affected wards especially male and female medical ward should be modified so that there are designated donning and doffing areas.
3. Appropriate and proper use of Personal Protective Equipment (PPE) including correct donning and doffing should be regularly reinforced by the hospital IPC team and the RRT.
4. All staff in the hospital must wear appropriate PPE for their level of risk of exposure which includes cleaning and security services. The hospital should consider deploying a dedicated team for enforcement of correct use of PPE. The hospital management should engage the private cleaning and security companies to ensure adequate and appropriate PPE for all staff
5. Arrangements should be made to decongest the affected wards so that there is sufficient distance between patients and to allow hospital staff to attend to patients with minimal risk of infection
6. The IPC team should organize random monthly audits of IPC practices and use of PPE with feedback to the affected wards.

#### 2.3.8. Initiate or Maintain Surveillance

Surveillance of COVID-19 cases was continued beyond 3 months reported in this paper. The different wards continued to report confirmed COVID-19 cases to the IPC team.

#### 2.3.9. Communicate Findings

The findings of the investigation were shared with the hospital management including nursing managers of the various wards. Key findings were also communicated with the DHMT and the Ministry of Health and Wellness (MOHW).

## 3. Discussion

The objectives of this outbreak investigation were firstly to describe person, place, and time (PPT) of COVID-19 cases in PMH and to determine measures to control and prevent further transmission of the virus within the facility. This was the first outbreak investigation of COVID-19 in PMH since the pandemic began. In this study, more than half of the participants were patients. This proportion is different to that observed in St Augustine hospital's COVID-19 outbreak in South Africa where the healthcare workers were twice the number of patients [7]. In a German hospital, an almost equal number of patients and healthcare workers were confirmed COVID-19 cases in a hospital outbreak [8]. Similarly, a health facility in Washington found a similar representation of patients and healthcare workers in its outbreak investigation [9]. The proportionality of COVID-19 outbreak between patients and staff in a hospital setting can be reciprocal with healthcare workers being the most likely drivers of transmission as they attend to different patients.

Further examination of the findings showed a median age of 35 years with a wide range for all participants (0–86 years). This appears to be influenced by the extreme ages of patients seen in PMH. In contrast, Jin et al. found the median age of patient diagnosed with COVID-19 to be 62 years in China [10]. The age range for healthcare workers, which is largely influenced by the policy of retirement age being set at 60 years in Botswana, was between 19 and 63 years [11]. The participants gender profile both overall and in subgroups of patients and healthcare workers were predominantly female in this investigation. This is similar to the findings in St. Augustine hospital in South Africa [7]. The prevalence between males and females is known to be comparable; however, there is a higher risk for adverse outcomes and mortality in male patients [10]. However, the observed finding in this investigation may be a reflection on the generally low health seeking behavior tendencies seen in men. Mthembu described that more than 70% of men in South Africa do not engage with healthcare services when they are sick [12]. Other studies have found a similar pattern of poorer health seeking behaviour among males than females in Botswana [13]. Female medical ward, emergency department, and dental unit were the most affected wards. This could be explained in part by the high frequency of predisposing aerosol-generating procedures in these departments. These procedures include endotracheal intubation and noninvasive ventilation [14]. It was expected that the emergency department would have a large number of cases, as it is the hospital entry point.

When COVID-19 was declared a pandemic in March 2020, several countries had reported confirmed cases. Early outbreak investigations were conducted in these countries. Investigators in Germany investigated a COVID-19 outbreak related to an index case that had come from China [15]. They reported 16 subsequent cases in 4 transmission generations. The reported median incubation period was 4 days, and in one instance, transmission occurred in the presymptomatic period. Outbreaks of COVID-19 have been reported in various health facilities across the world. In a nursing home in Illinois, United States of America, 26% of residents had confirmed COVID-19 infection [16]. In a living community for older adults in Seattle, Washington, 3.8% of residents and 3.2% of staff were found to have COVID-19 [9]. In another Washington residence facility, there were 167 confirmed cases of COVID-19 [17]. Of these, 101 were residents and 50 were healthcare workers. Most of the confirmed cases had symptoms consistent with COVID-19, but seven of the cases were asymptomatic.

COVID-19 outbreak investigations have been done in hospital settings. Hale et al. investigated an outbreak of COVID-19 in an American hospital food service workers after one person tested positive and was found to have continued to work while symptomatic [18]. There were 10 staff members who tested positive from the asymptomatic mass testing. None of the 8 staff members who were classified as close contacts tested positive for COVID-19. A COVID-19 outbreak investigation was done in Toronto, Canada, after 2 patients tested positive in April 2020. The investigation revealed COVID-19 infection in 4.6% of hemodialysis patients and 12% of staff members. Of these, 55% were asymptomatic at the time of testing while 32% were asymptomatic throughout the follow-up period [19]. Hospital acquired COVID-19 infections have been documented in the literature. In a British study, 15% of COVID-19 cases were nosocomial [20]. Another nosocomial COVID-19 outbreak was reported in a Korean hospital [21]. An adult tested positive after close contact with a pediatric confirmed case. The secondary patient's daughter later also tested positive.

The epicurve for the month of August 2020 showed a 3-day interval from the first confirmed COVID-19 case to the second patient. This was followed 7 days later by the 3 first confirmed cases in healthcare workers whose peak incidence was then five days later. These finding are consistent with the average pooled incubation period for SARS-CoV-2 which is 6 days [22]. These findings are also suggestive of directionality of transmission from patients to healthcare workers, although community transmission could not be ruled out. The subsequent decline in cases was due to the reemphasis of improved adherence and compliance to IPC measures that the RRT implemented. The challenge of seeing some spike increases in cases instead of a consistent decline may be due to pandemic response fatigue among the healthcare workers which was addressed with administrative measures to minimize or eliminate it.

The hospital managed to control the COVID-19 outbreak using IPC measures in addition to administrative controls to interrupt the transmission of the virus in the hospital. These included restricted access, restricted movement, dedicated staff to COVID-19 suspect patients, and confirmed cases awaiting transfer to the designated COVID-19 facility. The administrative control implemented via a policy enforced by the RRT to test every patient due for admission was crucial in limiting exposure of patients within PMH who are susceptible to COVID-19, thereby minimizing the burden of the outbreak.

### 3.1. Limitations

The investigation had some limitations. First, the patients' outcomes could not be confirmed for most patients since they were transferred to a designated COVID-19 isolation centre. Second, this report relies on the line lists prepared from the infection control team reports. Therefore, there were limited variables for analysis. Furthermore, the infection control team had to depend on reports of cases from the wards. It was therefore inevitable that some cases would have been missed.

Despite these limitations, this was a critical outbreak investigation of COVID-19 in a referral hospital in Botswana. The study demonstrated that public health interventions such as IPC measures could control outbreaks effectively within hospitals in Botswana.

## 4. Conclusion

The COVID-19 investigation in Princess Marina Hospital revealed an outbreak starting at the beginning of August 2020 and affecting patients and healthcare workers. Most wards in the hospital were affected, but the female medical ward, dental ward, and emergency department reported the most cases in the investigation period. Several control measures were implemented including reinforcing recommended IPC practices. The outbreak investigation was critical for understanding the outbreak and informing the most appropriate interventions.

## Figures and Tables

**Table 1:** Characteristics of all participants (*n* = 193).

Variable	Number (%)
Age, median (IQR)	35 (28–46)
Age, range	0–86
Age category	
Under 25	32 (16.6)
26–35	62 (32.1)
36–45	46 (23.8)
46–55	25 (13.0)
56–65	12 (6.2)
Over 65	11 (5.7)
Sex	
Female	154 (79.8)
Male	37 (19.2)
Not documented	2 (1.0)
Category	
Patient	110 (57.0)
Healthcare worker	83 (43.0)
Ward	
Antenatal ward	5 (2.6)
Dental department	8 (4.1)
Emergency department	24 (12.4)
Female medical ward	39 (20.2)
Female surgical ward	7 (3.6)
Gynecology ward	17 (8.8)
Labor ward	9 (4.7)
Male medical ward	5 (2.6)
Male surgical ward	5 (2.6)
Pediatric medical ward	10 (5.2)
Postnatal ward	7 (3.6)
Pediatric surgical ward	5 (2.6)
Others	52 (26.9)

**Table 2:** Characteristics of cases (patients and healthcare workers).

Variable, number (%)	Patients (*n* = 110)	Healthcare workers (*n* = 83)
Age, median (IQR)	35 (27–49)	36 (29–44)
Age, range	0–86	19–63
Age category		
Under 25	21 (19.1)	11 (13.3)
26–35 years	33 (30.0)	29 (34.9)
36–45 years	22 (20.0)	24 (28.9)
46–55 years	10 (9.1)	15 (18.1)
56–65 years	10 (9.1)	2 (2.4)
Over 65 years	11 (10.0)	0 (0)
Sex		
Female	87 (79.1)	67 (80.7)
Male	23 (20.9)	14 (16.9)
Not documented	0 (0)	2 (2.4)
Ward		
Antenatal ward	3 (2.7)	2 (2.4)
Emergency department	20 (18.2)	4 (4.8)
Female medical ward	23 (20.9)	16 (19.3)
Female surgical ward	4 (3.6)	3 (3.6)
Gynecology ward	16 (14.5)	1 (1.2)
Labor ward	7 (6.4)	2 (2.4)
Male medical ward	5 (4.5)	0 (0.0)
Male surgical ward	3 (2.7)	2 (2.4)
Pediatric medical ward	8 (7.3)	2 (2.4)
Postnatal ward	7 (6.4)	0 (0.0)
Pediatric surgical ward	3 (2.7)	2 (2.4)
Dental department	0 (0.0)	8 (9.6)
Others	11 (10.0)	41 (49.4)

## Data Availability

Data are available on request to the corresponding author.
